# Upon Improving the Performance of Localized Healthcare Virtual Assistants

**DOI:** 10.3390/healthcare10010099

**Published:** 2022-01-04

**Authors:** Nikolaos Malamas, Konstantinos Papangelou, Andreas L. Symeonidis

**Affiliations:** 1 Gnomon Informatics S.A., 570 01 Thessaloniki, Greece; konstantinospapangelou@gmail.com; 2 School of Electrical and Computer Engineering, Aristotle University of Thessaloniki, 541 24 Thessaloniki, Greece; symeonid@ece.auth.gr

**Keywords:** chatbot, virtual assistant, Rasa, ehealhtcare

## Abstract

Virtual assistants are becoming popular in a variety of domains, responsible for automating repetitive tasks or allowing users to seamlessly access useful information. With the advances in Machine Learning and Natural Language Processing, there has been an increasing interest in applying such assistants in new areas and with new capabilities. In particular, their application in e-healthcare is becoming attractive and is driven by the need to access medically-related knowledge, as well as providing first-level assistance in an efficient manner. In such types of virtual assistants, localization is of utmost importance, since the general population (especially the aging population) is not familiar with the needed “healthcare vocabulary” to communicate facts properly; and state-of-practice proves relatively poor in performance when it comes to specialized virtual assistants for less frequently spoken languages. In this context, we present a Greek ML-based virtual assistant specifically designed to address some commonly occurring tasks in the healthcare domain, such as doctor’s appointments or distress (panic situations) management. We build on top of an existing open-source framework, discuss the necessary modifications needed to address the language-specific characteristics and evaluate various combinations of word embeddings and machine learning models to enhance the assistant’s behaviour. Results show that we are able to build an efficient Greek-speaking virtual assistant to support e-healthcare, while the NLP pipeline proposed can be applied in other (less frequently spoken) languages, without loss of generality.

## 1. Introduction

Virtual assistants are software programs designed to imitate a conversation with a human and perform specific, often repetitive, tasks. They have a long history with ELIZA [1] dating back to 1966. After that, rule-based approaches dominated, leading to better systems but still with limited flexibility [2]. The recent advances in Natural Language Processing (NLP) and Machine Learning (ML) have motivated the extension of these systems besides the traditional areas of application (e.g., customer service, marketing) and have opened new directions.The recent advances in Natural Language Processing (NLP) and Machine Learning (ML) have accelerated their development and motivated the extension of such systems besides the traditional areas of application (e.g., customer service, marketing) opening new directions. A detailed history of their development along with some key milestones can be found in Tudor Car et al. [2]. In this work, we focus on e-healthcare, an area that can benefit significantly from such conversational models.

The application of virtual assistants in e-healthcare comes with many challenges that are related to the technology, but also its acceptability by the end-users. It is common knowledge that there is a hesitancy regarding the use of chatbots in healthcare [3], while a key factor is whether the user can trust the assistant’s ability to provide valid advice [4]. Despite the identified challenges, e-healthcare remains a core area of interest and a variety of commercial and experimental English-speaking assistants have been developed focusing on different sub-domains and tasks [5]: they can be used to check symptoms, provide well-being recommendations, retrieve information, track activities and habits and perform teleconsultation, just to just name a few.

An important area of interest, where we focus our study, is home healthcare and self-management/monitoring. Such English-speaking commercial assistants are already available, such as Ada [6] and Youper [7]. Ada is a healthcare companion mobile application for patients at home. It can be used for health monitoring and improvement as it checks whether patients are following their prescriptions and sticking to their recommended lifestyle via a textual messaging channel. On the other hand, Youper is a self-help application focused on mental health assessment. It offers AI-employed therapy sessions to efficiently check and track users’ emotional status and recommend relevant exercises through a textual interface. In the same manner, Fitzpatrick et al. [8] developed Woebot, an agent that people suffering from depression and anxiety can chat with throughout the day. Preliminary results of a 2-week period Woebot usage indicate a significant reduction of anxiety in patients. In addition, Bickmore et al. [9] created a virtual coach to motivate elderly people to walk more. A positive impact was noted in most cases during the first 2 months of use, improving the health and well-being of users. Finally, a personal health condition tracker was presented in Ferguson et al. [10]. This prototype offers a voice-based interface for patients to gather information daily as day-to-day monitoring is crucial for patients who suffer from diseases such as heart diseases and chronic pain. These assistants are summarized in Table 1, while more systematic reviews of assistants in the e-healthcare domain can be found in other studies, such as Tudor Car et al. [2] and Isern et al. [11]. Although the above-mentioned approaches are interesting, they do not support localization efficiently, thus non-native English speakers may be more reluctant to use them. Additionally, they do not focus on building a remote, yet stable relationship with physicians.

In this work, we address the above challenges and focus on building a virtual assistant capable to automate a set of tasks associated with a person’s well-being and fostering the relationship with their physicians. We are particularly interested in scenarios that can help the elderly population access a physician; in these cases, the need to access may be frequent but in-person visits may be difficult. Additionally, we are interested in allowing users (and their relatives) to monitor their treatment plans assigned by their physicians. Since localization is even more important in this population segment, a key objective of our work is to build an assistant that can be adapted to less frequently spoken languages and in our case the Greek language. The majority of those low-resource languages lack of datasets or models and have usually not been thoroughly evaluated [12]. Moreover, the use of multilingual models cannot yet ensure similar performance with monolingual ones [13], as each language has its own unique characteristics that should be considered. Consequently, we cannot use pipelines implemented in English as-is for similar reasons.

In this context, the proposed NLP pipeline for implementing an efficient assistant involves solving various NLP tasks in order to process the user’s utterance and extract useful information. These two factors dictate employing a conversational infrastructure appropriately adjusted to less frequently spoken languages, while also building rule-based or ML methods for delivering the expected virtual assistant functionality. In order to build an ML-based assistant firstly we need to extract meaningful features from the raw text. This is performed by a pipeline of various components that perform the necessary NLP tasks. For the implementation of our system we will focus only on a few required steps. First is the tokenization process, where each input phrase is split into smaller units (e.g., words or subwords). Then follows the feature creation, where each token can be represented by multiple numeric vectors expressing for example frequency of occurrence or expressing its meaning in a multidimensional space where vector distances indicate similarity between the corresponding tokens. In the following sections we will describe these in more detail along with the used tools. As already mentioned, the proposed NLP pipeline can be seen as a recipe, easily applied to other languages, considering that the appropriate language-specific modifications are made (i.e., create a new corpus with intents and examples, select a language-specific tokenizer, compare various language-specific embeddings and possibly consider the features that will be provided to the classifiers).

Within the context of this work we contribute to the following axes: (a) we present what is –to the best of our knowledge– one of the first Greek e-healthcare virtual assistants, (b) we evaluate the performance of the Rasa framework by employing state-of-the-art word representations in Greek, such as Greek-BERT [14], to better adapt to the nuances of the language and, (c) we assess the performance of the developed assistant based on conversations from several test users. The rest of the paper is structured as follows: Section 2 describes the implemented assistant scenarios, the used ML pipeline and the followed evaluation process. Next, Section 3 presents the experimental results and discusses the outcomes. Finally, Section 4 summarizes the key aspects of our work and Section 5 concludes the paper.

## 2. Materials and Methods

### 2.1. Technology Decisions

Based on our analysis in [15], we build our pipeline on top of Rasa [16], a robust and scalable open-source framework that comes with configurable components that cover most functionalities of an NLP pipeline (e.g., tokenization, n-gram creation, entity extraction). In fact, Rasa can achieve similar performance with commercial systems like Google Dialogflow or Microsoft LUIS as studied in [17,18,19,20], while offering the advantages of an open-source end-to-end system (e.g., self-hosted, secure, scalable, fully disclosed) [21]. Applications of Rasa can be found on various domains, as a Spanish question answering agent of the football domain [22], a university campus information system [23] and a Vietnamese-speaking agent [24] among others.

Additionally, in order to properly support the envisioned functionality, our approach is built on top of eHealthPass™ (https://www.gnomon.com.gr/ehealthpass/, Access Date: 1 November 2021), a complete e-health platform that handles appropriately all relevant user and doctor information, while caters for privacy and consent management. eHealthPass™allows self-management as well as patient-doctor collaboration and integration of various wellness-related IoT devices.

For our implementation to be efficient we need to address two main challenges; firstly, adjust any language-specific components to the Greek language and secondly, further optimize the Rasa infrastructure considering the key tasks of intent and entity recognition. The first task refers to identifying what the user wants to achieve and the second is the problem of extracting useful information for performing the corresponding task. As the developed virtual assistant is task-oriented, each user expression triggers the most appropriate response or action among a set of predefined scenarios. The supported scenarios can be expressed as a sequence of intents and responses/actions. We argue that if the correct intent is identified from a user’s utterance and this is among the supported scenarios, this will initiate the correct sequence of events allowing the user to eventually complete the task. Therefore, intent recognition is closely related to task completion and for this reason, we will focus our evaluation mainly on this task. We adopt language-specific components to integrate the Greek language in Rasa, while we employ several methodologies for representing sentences as feature vectors for the ML models. Next, we evaluate different ML classifiers on our task-specific dataset and, finally, we share a version of our assistant with users and report the preliminary results regarding the task of intent recognition. Again, we stress that the pipeline proposed can be easily applied to other languages, given that targeted language-specific modifications are made.

### 2.2. Supported Scenarios

**Figure 1:**
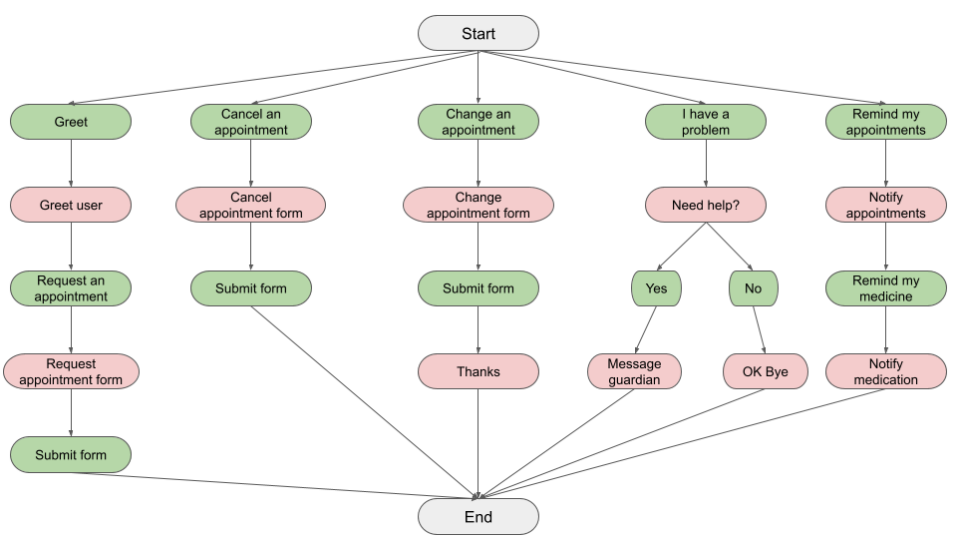
Examples of supported scenarios. The green boxes represent user intents and the red the assistant’s responses or actions.

Pivotal to the usability and performance of a virtual assistant is the proper definition of the user’s *intents*, the *entities* extracted, and the *actions* to perform when a user intent is identified. Given the scope of our virtual assistant, we focus on four broad categories related to e-healthcare: (a) doctor’s appointment management, (b) medication management, (c) emergency notification and, (d) supporting functionalities. An overview of some scenarios is provided in Figure 1 with all the necessary intents and actions depicted.

Through the developed assistant the user can request, cancel and/or change a doctor’s appointment, as well as get informed about the appointments they have already booked. Each of these tasks can be recognized by the assistant when the corresponding intent is identified correctly. In order to perform some of these tasks, the user needs to provide some information, which are the entities the agent needs to identify. In particular, for booking an appointment the relevant entities are the doctor’s name (perhaps their specialty) and the date and time of the appointment. Similarly, the user needs to provide this information when canceling or changing an appointment. Additionally, the user can be notified about their booked appointments by asking the assistant and can also provide a specific period for which they are interested in learning their appointments. A notification mechanism has also been developed to notify the user about their booked appointment at a predefined time interval (e.g., the previous day). This way the user can either confirm the appointment or ask for change or cancellation. This is, however, not triggered by an intent and hence will not be considered in our evaluation. We can therefore identify four intents for the aforementioned scenarios: request, cancel, and change an appointment as well as ask about an already booked appointment. For each of these four intents, we create a corpus of sentences, each one describing different ways with which a user can perform the corresponding task. Each of these sentences will be an example that will be fed to the ML model to get a prediction. To introduce variability between the examples, we consider many different ways with which a user can e.g., request an appointment, by providing the doctor’s name, their specialty, and/or the date or time they wish to book the appointment. The number of examples per intent is shown in Table 2.

Additionally, the user can be informed about their medication plan either upon request or via a notification mechanism (similarly to the appointment notification procedure). Specifically, the user can be informed about their daily medication by asking the assistant and triggering the corresponding intent (“Information for drugs”). Also, the user can be reminded, e.g., at the beginning of each day, about the medication they have to take. After that, they will be prompted to verify whether they have taken their medication or not. In case they have not taken their medication they are asked to provide more information and their response is saved and can be forwarded to the doctor or the patient’s relatives. Such reminder schemas provide an efficient medium for collecting useful information about the patients’ condition remotely and can potentially handle other aspects of their treatment plan (such as daily exercise, diet, etc.). This information is available via the eHealthPass™platform, which additionally allows connection with external devices for better health monitoring. It should be noted that this reminder schema is developed to be triggered via automated HTTP requests instead of user expressions, hence these cases are not considered in the described dataset and the evaluation of the ML pipeline.

The virtual assistant also provides the ability to notify a selected emergency contact in case of an unexpected problem, such as severe pain or fall. When this particular intent (“Problem”) is triggered, the assistant first queries on the users’ condition (“I am fine”, “I am hurting”, etc.). Then the user may notify their contacts (e.g., relatives) by sending a message via the eHealthPass™platform containing the user’s utterances.

In order to create a better user experience, we have included some supporting intents to handle the conversation flow. For example, the user may be prompted to affirm or deny the appointment before it is booked. These will be captured by the corresponding intents. While booking an appointment, the user may also need to provide information about the appointment which by itself corresponds to a different intent (“Inform”). The user might also want to interrupt the procedure, which is again captured by a different intent (“Stop”). Finally, we have included intents that support general conversation (“Greet”, “Thanks”, “Ask functions”).

The aforementioned corpus is of a not large size as it is hand-designed and created in order to represent as many real examples as possible. This is common practice when creating virtual assistants, especially with the Rasa framework [15,22], where the expansion of the NLP corpus is an iterative process of collecting, reviewing and adding new data from real conversations. Hence, this dataset can be considered as an initial one on top of which all progress will occur. In addition, the designed intents are of varying size and similarity. Regarding the example size, the intents that correspond to appointment management can include rather long expressions, while others include only short expressions, even one word long (e.g., “Affirm”, “Deny”, etc.). As for the intent similarity, those that are related to appointment management, as well as some of the supporting intents, may differ with respect to only one or two words. Hence, we believe this corpus to be representative of various scenarios and can test the limitations of the designed system.

As described above, a key factor in the success of the proposed virtual assistant behavior is the correct identification of the corresponding intent. If a user’s intent is not identified correctly, then the wrong action might be triggered. To this end, we focus on how this problem can be tackled using different ML and NLP choices. We also evaluate the entity recognition problem, which can be tackled to some extent using off-the-shelf tools.

### 2.3. Proposed ML Pipeline

In this section we discuss the ML pipeline followed, along with the components employed, the features extracted and the evaluation mechanism defined.

#### 2.3.1. Pipeline

**Figure 2:**
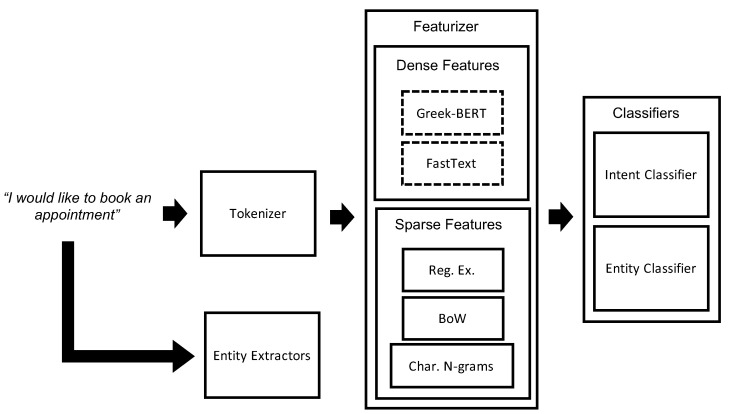
Description of the pipeline designed. Dashed boxes indicate that when dense features were used we chose one of the two options.

Given a sentence, the process we followed is described in Figure 2. Firstly, the input was tokenized into words and/or character n-grams (here we used the former). Then, for each token, as well as for the whole sentence, various feature vectors were extracted. These were subsequently used to perform the relevant classification tasks. The sentence was also passed to various extractors in order to identify useful entities.

In order to tokenize the sentences, Spacy [25] was used, a popular NLP framework that supports the Greek language (https://github.com/eellak/gsoc2018-spacy, Access Date: 1 November 2021). Once the sentences were tokenized, various featurizers were employed to extract meaningful features. In Rasa, features are categorized as sparse and dense. Sparse features were created entirely from our dataset, presented in Section 2.2, while dense features were pre-trained word representations obtained from various sources. Regarding the sparse representations of the sentences, we employed three featurizers. The first extracted pre-specified regular expressions (such as numbers, date, and time in specified formats), as well as common values of some entities. The second generated character n-grams, while the third built a bag-of-words representation.

Regarding the dense representations, we employed two models trained in the Greek language. Greek-BERT [14] is a model that is similar to BERT-BASE [26] and is trained on the Greek parts of three corpora, namely articles from Wikipedia, the European Parliament Proceedings Parallel Corpus [27] and OSCAR [28]. Since BERT is already supported by Rasa as a dense featurizer we included it without any modifications. We additionally explored an alternative option using FastText embeddings [29] suited for the language of our application. These have been trained on a corpus derived from approximately 20M URLs in the Greek language [30,31,32]. The aforementioned dense representations that utilized pre-trained embeddings resulted in a feature vector for each word. These features were then used to build ML classifiers for identifying the intents and entities.

For intent classification, we employed a Support Vector Machine (SVM) classifier with polynomial and RBF kernels (which is also a built-in component in Rasa) and selected the optimal hyper-parameters via cross-validation by optimizing the weighted average of F1 scores calculated for each intent. The input for SVM was the average of word embeddings for each sentence. When using Greek-BERT we also considered the case of joint classification of intents and entities using DIET [33], a deep learning architecture trained on both sparse and dense features in order to optimize a joint loss function that accounts for both entity and intent recognition. Regarding entity classification, we used DIET, but we also extracted some key entities using Spacy and Duckling (https://github.com/facebook/duckling, Access Date: 1 November 2021). The former can be used to identify names and the latter timestamps. It is worth noting that in our application most useful entities take values from a pre-specified set, hence entity extraction can also be handled with rule-based methods. In Table 3 we report the details of the configurations we used to train the assistant for intent recognition. The difference between configurations 1 and 8 is the structure of DIET. The latter uses deeper networks with twice the number of layers, the embedding dimension, and the size of the hidden layers.

#### 2.3.2. Evaluation Framework

We evaluated different pipelines using repeated 5-fold stratified cross-validation. In particular, we repeated the cross-validation procedure 5 times, each time using a different partition of the data. We employed stratified cross-validation in order to ensure that each intent was represented in the training and testing data. We used the following widely used metrics:

(1) $$\begin{array}{c} Accuracy=\frac{TP+TN}{TP+FP+TN+FN} \end{array}$$

(2) $$\begin{array}{c} Precision=\frac{TP}{TP+FP} \end{array}$$

(3) $$\begin{array}{c} Recall=\frac{TP}{TP+FN} \end{array}$$

where TP, TN, FP and FN are the number of True Positive, True Negative, False Positive and False Negative records, respectively. These were calculated for each intent separately and then we used both the unweighted and weighted average. The first is simply the value of the metrics averaged over all intents, while the second weights the metric for each intent against the related number of examples. One should note that particularly for those intents with only a few examples precision may not be calculated. To this end, we used only the values for the samples where it is defined. We report the average value of the metrics and the standard deviation across all folds.

Furthermore, we focused on a configuration that provided good empirical results and trained a model on the entire dataset. We then shared our assistant with users familiar with programming who had no experience in the development of virtual assistants. They performed in total 10 unique sessions in a one-week period resulting in conversations of varying size and with different tasks. Their given guidelines were the scenarios the assistant supports in order to simulate a real-world environment as much as possible. Our primary objective was to extract preliminary findings in a real-world setting, while a more extensive evaluation would be an appropriate next step. To efficiently support those sessions, we used Rasa X (https://rasa.com/docs/rasa-x/, Access Date: 1 November 2021), an extension of the main Rasa framework that offers a chatting user interface to instantly interact with a deployed model. Each conversation included at least one of the following six scenarios: request an appointment, cancel an appointment, change an appointment, medication reminder, appointment reminder, and emergency. Our purpose was to evaluate how the model would perform on a separate test set created by the users’ interactions with the assistant. In the next section, we present the empirical results as well as the assistant’s performance in the task of intent recognition using the data collected from the users’ conversations.

## 3. Results

### 3.1. Intent Recognition Results

In Table 4 we report the accuracy at the task of intent recognition as well as the weighted and unweighted precision and recall. Interestingly, we find that using the DIET classifier with only sparse features shows better results than using the BERT features alone. Also, the SVM classifier trained on average word embeddings shows higher average accuracy compared to using DIET with BERT features. We note that DIET comes with many hyperparameters that can be tuned and an exhaustive search can be computationally demanding. We replace the BERT representations with FastText and find that the latter with an SVM classifier and RBF kernel shows good empirical results, comparable with the more complex architectures. Even though the differences between some configurations are small, we will now focus on the aforementioned configuration (configuration 7) which besides its performance, is also attractive due to its simplicity.

In Table 5 we report the average precision and recall for each intent separately. We observe that recognizing correctly certain intents is more challenging than others. A closer inspection shows that affirmation and denying can often be confused and the same holds for the intents that express that the user is fine and having a problem. We expect this is to be not only because of the few examples in our dataset but also because of the similarity of those sentences. For example, in our setting the distinction between sentences that indicate affirmation and denying is sometimes only due to one word. We note, however, that these intents can be expressed by most users in similar ways and performance in the training data (where they are classified correctly) is also important. Other intents such as those that correspond to requesting (or modifying/canceling) an appointment can be expressed in many different ways. For example, we can expect that a user might simply request an appointment by specifying a doctor, specialty, date or time, or combinations of those, hence resulting in a large number of possible combinations. Again by looking at the most often misclassifications, we find that similar intents where the sentences can only differ with respect to a few words can be confused. For example, the change of appointment is most often misclassified as requesting or canceling, while asking information about existing appointments can often be misclassified as requesting a new one.

### 3.2. Entity Recognition Results

Regarding the entity prediction task using DIET, we found that all studied approaches achieve good empirical results with the minimum observed average weighted precision and recall being 0.977 and 0.966 respectively. As we have already described, in our setting we are mainly interested in identifying doctors, specialties, and time entities. The first can also be handled by Spacy, the second by searching directly in the sentence, since we can expect a pre-specified set of specialties, and the latter using Duckling. To this end, the DIET classifier is mainly used to support those components. For completeness of the presentation, we report in Table 6 the results of DIET for one of the most important entities in our implementation.

### 3.3. User Evaluation

Finally, we shared an assistant trained on the entire dataset using configuration 7 to a number of users. We collected 10 conversations from users who performed at least one of the six key tasks. We then created a test set by labeling the sentences according to the corresponding intent. We filtered out duplicates, expressions that contained non-Greek words as well as expressions that did not correspond to intents that we have in our data. This resulted in a dataset with 142 sentences which can be considered as our test set. We found that the accuracy of the model on the task of intent recognition was 87.3%. We remind the reader that the average value observed in our previous experiment was 89.9%. We note that approximately 87% of the sentences are new and do not appear in our training data, even though some are similar and vary with respect to only a few words (77% if we ignore the intent “Inform” which captures different pieces of information a user might provide such as times and dates and therefore we can expect these sentences to be different).

In Table 7 we present the recall for the 6 intents that correspond to the 6 tasks our assistant performs. We find that the intents of requesting information for appointments and expressing a problem are the hardest to recognize correctly. Interestingly, if the intent “Information for appointment” is not recognized correctly it is likely classified as “Request an appointment” since that happened to 4 out of the 5 misclassifications. This was also observed in our previous experiments and could be attributed to the similarity of the sentences expressing these intents. In addition, the intent “Problem” was misclassified in two out of three cases as “I am fine”. This could be attributed to the size of the examples as they contain only few words, with some similarities between those. Experiments of larger scale would allow us to better understand such misclassifications. The average recall of intent recognition for the 6 key tasks is 81.25%.

We focused so far on the results using configuration 7 which we chose due to its simplicity and cross-validation results. However, in a real-world setting, we might not always observe the same performance. It would be interesting to explore what happens if we use some other models. We apply configurations 6 and 8 that use DIET and find that they achieve an accuracy of 87.3% and 83% respectively. These could also be considered as promising candidates for future developments and testing. Other aspects of the problem such as inference time, the confidence of the predictions, or placing higher importance on certain intents could also inform our final decision.

## 4. Discussion

In this work, we studied the problem of building a virtual assistant that can support less frequently spoken languages. The described procedure is rather general and could provide a recipe for building assistants in other languages with the appropriate modifications. Here, we focused on the Greek language and designed an assistant for supporting common tasks such as managing appointments and the user’s treatment plan. In this work, we presented a Greek ML-based virtual assistant designed to support common tasks in the healthcare domain. Our system builds on the Rasa framework and as such it follows the supported pipeline. Similar to previous works [23,24] we explore how this pipeline can be appropriately modified with some key differences being the language, the domain of application and the explored featurizers. Additionally, in this work we focus primarily on the ML components comparing both different classifiers and also embeddings in more detail. We also note that in this work we perform experiments with real users in order to explore both results in our dataset but also the generalization of our system in a real-world setting focusing on the ML tasks. Other studies using the Rasa framework have focused on quantifying user satisfaction [22] and this could be an interesting next step in our study.

We first described the supported tasks and the associated intents we would like to recognize. We then focused on the ML components one could use in such a setting and evaluated their performance at the tasks of intent and entity recognition. The first task will guide what action the assistant will perform and the second task provides useful information for completing this action. Overall, we found that taking the average of word embeddings and training an SVM classifier showed promising results. The DIET classifier also performed well when using either only sparse features or both sparse and dense features. Using components designed for the specific language, instead of multilingual ones, has shown that can improve the results at the above tasks [24]. In this work, we also focused on modifying the required components for our language of interest and observed promising results.

We then focused on a single configuration and explored what happens for each intent separately. We noticed that certain intents can be particularly challenging to identify correctly. This could be due to the limited number of examples and/or their similarity. Additionally, we observed that, as expected, most misclassifications are between intents that share common words. Their distinction is a challenging problem and could be the focus of future work. We note, however, that for certain intents we do not expect large variations between the different ways users can express them, allowing us to focus on those that may affect the supported scenarios to a larger extend.

Lastly, we evaluated selected configurations in a separate test set comprised of sentences derived from users who interacted with a version of the assistant. Here, we focused on the recall of intent classification. In particular, we expect that if the user expresses a particular intent and this is classified correctly then the correct action will be performed and therefore the user will eventually complete their task. Similar to our previous findings, we found that certain tasks can be more challenging. We note that the development of an assistant is a time-consuming procedure that requires repeating the training of a (new) model and collecting new data ideally using interactions with real users.

This is to the best of our knowledge one of the first studies that evaluates an ML-based Greek virtual assistant in the domain of healthcare. Since the proposed approach builds on the Rasa framework it has the advantage that can be adapted to other languages while it offers various built-in components. This study also offers a more detailed analysis of the NLP pipeline using both hand-designed and real-world data. A limitation of this study is the scale of the real-world experiment. Here we performed a preliminary study with few participants/conversations, however, a larger study could allow us to identify new directions of improvement. To this end, we could also explore other aspects that are related to the assistant’s functionality such as the time required for completion of tasks that require multiple steps, e.g., booking an appointment. Another aspect we did not explore is user satisfaction as can be measured e.g., using questionnaires [22].

We would also like to highlight some potentially interesting future directions. As we described, the DIET classifier comes with many hyperparameters that control the complexity of the model, hence a more extensive evaluation of its configuration could provide some benefit. We remind the reader that when using the SVM classifier the sentence representation was derived by averaging the word embeddings. However, we note that the literature is vast with approaches for getting sentence representations from word embeddings using different combinations of those or by building some model (e.g., [34,35,36]). Since the choice of sentence embeddings can play a role in the performance of the model, the evaluation of such approaches at the task of intent recognition for building a virtual assistant would be an interesting future direction.

## 5. Conclusions

In this work, we studied the problem of building a virtual assistant in order to support tasks related to a person’s well-being. Using the Rasa framework we focused on the design of a pipeline that can support less frequently spoken languages. The described procedure is rather general and could provide a recipe for building assistants in other languages with the appropriate modifications. Here, we focused on the Greek language and designed an assistant for supporting some common tasks such as managing appointments with physicians and the user’s treatment plan. Results at the task of recognizing the user’s intent are promising, while a more extensive evaluation with more users would be a potential next step.

## Figures and Tables

**Table 1:** Summary of presented virtual assistants.

Virtual Assistant	Use Cases	Interface
Ada [6]	Health monitoring	Mobile text application
Youper [7]	Mental health assessment	Mobile text application
Fitzpatrick et al. [8]	Depression therapy	Mobile text application
Bickmore et al. [9]	Virtual coach	Tablet touch screens
Ferguson et al. [10]	Health condition tracker	Voice interface

**Table 2:** Supported intents.

Intent	Number of Examples
Request appointment	151
Change appointment	121
Cancel appointment	115
Inform	79
Information for appointment	75
Information for drugs	55
Affirm	30
Ask functions	25
Problem	22
Confirm suggestion	21
Deny	21
Greet	17
I am fine	17
Stop the procedure	11
Thanks	9

**Table 3:** Description of used configurations for the task of intent recognition.

Configuration	Description
1	DIET with sparse + BERT features
2	DIET with sparse features
3	DIET with BERT features
4	polynomial SVM with BERT features
5	polynomial SVM with FastText features
6	DIET with sparse + FastText features
7	RBF SVM with FastText features
8	DIET (modified) with sparse + BERT features

**Table 4:** Average and standard deviation (in the parenthesis) for each metric at the task of intent classification. For the precision and recall the first line corresponds to weighted and the second to unweighted average.

Configuration	Accuracy	Precision	Recall
1	0.883 (0.025)	0.883 (0.025)	0.883 (0.025)
		0.806 (0.048)	0.733 (0.052)
2	0.894 (0.027)	0.899 (0.028)	0.894 (0.027)
		0.819 (0.053)	0.777 (0.055)
3	0.749 (0.035)	0.760 (0.034)	0.749 (0.035)
		0.720 (0.056)	0.639 (0.058)
4	0.877 (0.018)	0.887 (0.017)	0.877 (0.018)
		0.854 (0.039)	0.787 (0.040)
5	0.889 (0.021)	0.899 (0.021)	0.889 (0.021)
		0.843 (0.051)	0.793 (0.055)
6	0.901 (0.022)	0.906 (0.022)	0.901 (0.022)
		0.837 (0.044)	0.788 (0.044)
7	0.899 (0.021)	0.907 (0.020)	0.899 (0.021)
		0.866 (0.037)	0.811 (0.040)
8	0.900 (0.023)	0.912 (0.021)	0.900 (0.023)
		0.844 (0.044)	0.780 (0.052)

**Table 5:** Average precision and recall for each intent using configuration 7.

	Intent	Precision	Recall
1	Information for drugs	0.990 (0.034)	0.945 (0.062)
2	Change appointment	0.965 (0.037)	0.966 (0.033)
3	Information for appointment	0.938 (0.058)	0.941 (0.060)
4	Cancel appointment	0.968 (0.028)	0.940 (0.045)
5	Confirm suggestion	1.00 (0.000)	0.898 (0.168)
6	Request appointment	0.913 (0.049)	0.949 (0.037)
7	Inform	0.857 (0.064)	0.949 (0.058)
8	Ask Functions	0.785 (0.146)	0.744 (0.165)
9	Thanks	0.960 (0.195)	0.840 (0.272)
10	Greet	0.866 (0.223)	0.646 (0.269)
11	Problem	0.677 (0.181)	0.755 (0.186)
12	Affirm	0.727 (0.164)	0.793 (0.158)
13	Deny	0.846 (0.183)	0.574 (0.286)
14	I am fine	0.624 (0.285)	0.590 (0.274)
15	Stop the procedure	0.884 (0.227)	0.633 (0.294)

**Table 6:** Classification results for the entity “doctor”.

Configuration	Precision	Recall
1	0.995 (0.013)	0.911 (0.059)
2	0.960 (0.042)	0.829 (0.061)
3	0.994 (0.013)	0.914 (0.080)
4	0.994 (0.014)	0.906 (0.061)
5	0.995 (0.015)	0.912 (0.061)
6	1.00 (0.000)	0.909 (0.067)
7	0.998 (0.008)	0.914 (0.071)
8	0.983 (0.025)	0.936 (0.059)

**Table 7:** Preliminary results for the intent prediction problem in the test data.

	Intent	Recall
1	Request appointment	100% (11/11)
2	Change appointment	100% (9/9)
3	Cancel appointment	100% (3/3)
4	Information for appointment	50% (5/10)
5	Information for drugs	87.5% (7/8)
6	Problem	50% (3/6)

## Data Availability

Not applicable.
